# Genome-Wide CRISPR Screens Identify ABCG2-Mediated Drug Resistance to the Threonine Tyrosine Kinase (TTK) Inhibitor CFI-402257 in Breast Cancer

**DOI:** 10.3390/ijms27062665

**Published:** 2026-03-14

**Authors:** Kelsie L. Thu, Soode Jafari, Jennifer Silvester, Jennifer Cruickshank, Isabel Soria-Bretones, Kelsey Hodgson, Chantal Tobin, Jillian Haight, Asa P. Y. Lau, Tessa Bray, Drew Wakeham, Mark R. Bray, Tak W. Mak, David W. Cescon

**Affiliations:** 1 Keenan Research Centre for Biomedical Science, St. Michael’s Hospital, Unity Health Toronto, Toronto, ON M5B 1X3, Canada; 2 Department of Laboratory Medicine and Pathobiology, University of Toronto, Toronto, ON M5S 3K3, Canada; 3 Princess Margaret Cancer Centre, University Health Network, Toronto, ON M5G 2C4, Canada; 4 Department of Immunology, University of Toronto, Toronto, ON M5S 3K3, Canada; 5 Department of Medical Biophysics, University of Toronto, Toronto, ON M5G 2C4, Canada; 6 Institute of Medical Science, University of Toronto, Toronto, ON M5S 3K3, Canada

**Keywords:** CRISPR screen, breast cancer, ABCG2, drug resistance, CFI-402257, luvixasertib

## Abstract

CRISPR screens are a powerful functional genomics approach for identifying genes that confer sensitivity and resistance to anti-cancer therapies. CFI-402257 (luvixasertib, 2257) is a small molecule inhibitor of threonine tyrosine kinase (TTK), a promising therapeutic target in genomically unstable cancers due to its critical role in establishing the spindle assembly checkpoint (SAC) during mitosis. To inform its ongoing development and evaluation in clinical trials, we sought to use CRISPR activation (i.e., gain of function) screens to identify cellular mechanisms of resistance to 2257 in models of triple-negative breast cancer (TNBC). In vitro screens conducted in two TNBC cell lines nominated ABCG2 as the top resistance-conferring gene in both models. Validation studies assessing clonogenic survival and apoptosis confirmed that ABCG2 overexpression enhanced TNBC resistance to 2257 in vitro, while knockdown enhanced sensitivity. These findings suggest that 2257 is a substrate of ABCG2’s drug efflux activity. However, overexpression of ABCG2 failed to confer resistance to 2257 in TNBC xenografts grown in mice and treated with a moderately active dose and schedule. Our results highlight the potential impact of drug transporters in in vitro CRISPR screens and the importance of confirming the relevance of drug response mechanisms identified in cultured cells using in vivo models that recapitulate drug pharmacokinetics and pharmacodynamics.

## 1. Introduction

The spindle assembly checkpoint (SAC) is a cell cycle checkpoint that halts mitotic progression until all chromosomes are properly attached to microtubules of the mitotic spindle [1]. Its activation prevents dividing cells from premature progression to anaphase, which can result in severe chromosome segregation errors and aneuploidy in the daughter cells produced. As such, the SAC has a critical role in maintaining genomic integrity. Chromosomal instability (CIN) is a hallmark of cancer cells characterized by a high rate of chromosome missegregation [2,3]. Although CIN creates genetic diversity that fuels tumor evolution and disease progression, it also renders cancer cells hyperdependent on SAC function to survive. As a result, the SAC has emerged as a promising therapeutic target in genomically unstable cancer cells [4,5]. Research in preclinical models has shown that inhibiting kinases that regulate the SAC can induce cancer cell-selective death in several tumor types [4].

One such kinase is threonine tyrosine kinase (TTK), also known as monopolar spindle 1 (MPS1). TTK activity activates the SAC during mitosis when kinetochores that are unattached to spindle microtubules are present [1]. Overexpression of TTK is common in several malignancies and is associated with poor prognosis of cancer patients [5]. Given its therapeutic potential in cancer, numerous TTK inhibitors have been developed, and several small molecules targeting TTK are being evaluated in clinical trials [6,7]. The premise for these drugs is that inhibiting TTK to abrogate SAC function will potentiate CIN to intolerable levels in cancers with high basal instability. CFI-402257 (2257) is one of the most potent and selective TTK inhibitors available and has demonstrated encouraging anti-cancer activity in preclinical models of glioblastoma, mesothelioma, and breast, lung, and liver cancers [8,9,10,11,12,13]. Multiple phase I and II trials are investigating 2257 in patients with advanced solid tumors, including breast cancer patients. Based on preclinical evidence [8], 2257 received fast-track designation by the FDA for the treatment of ER+/HER2- breast cancer patients whose disease progresses after treatment with CDK4/6 inhibitors combined with hormone therapy. To guide its ongoing clinical development and utility for treating breast cancer, a better understanding of the molecular mechanisms underlying tumor response to 2257 is desired.

We previously used CRISPR/Cas9 loss-of-function (LOF) screens to investigate 2257 response mechanisms in triple-negative breast cancer (TNBC) cells, which revealed that delaying anaphase progression confers resistance to TTK inhibition [14]. For this study, we reasoned that response to 2257 may also be driven by gene gain-of-function (GOF). To address this idea, we sought to discover mechanisms of resistance to 2257 using CRISPR activation (i.e., GOF) screens in TNBC models. Our screens and validation studies identified that overexpression of the ATP-binding cassette transporter, ABCG2 (also known as Breast Cancer Resistance Protein, BCRP), rendered TNBC cells resistant to 2257 in vitro. ABCG2 is a well-established driver of multidrug resistance [15], and our work nominated 2257 as a substrate of this drug-efflux pump. Further in vitro experiments supported this concept, as pharmacologic and genetic inhibition of ABCG2 conferred sensitivity to 2257 in TNBC cells. However, overexpression of ABCG2 in TNBC xenografts failed to confer resistance to 2257 in vivo, suggesting that ABCG2 is unlikely to contribute to 2257 resistance in patients. These findings indicate that candidate mediators of drug response identified using in vitro CRISPR screens do not always translate to in vivo conditions, highlighting the importance of validating screen hits in animal models to confirm their clinical significance. Furthermore, our work demonstrates that pharmacologic mediators independent of a drug’s mechanism of action can limit insights gained from functional genomic screens to identify response mechanisms. This could inform improved rational design of screening libraries.

## 2. Results

### 2.1. CRISPR Screens Reveal ABCG2 as a Putative Mediator of TNBC Response to 2257

**Figure 1:**
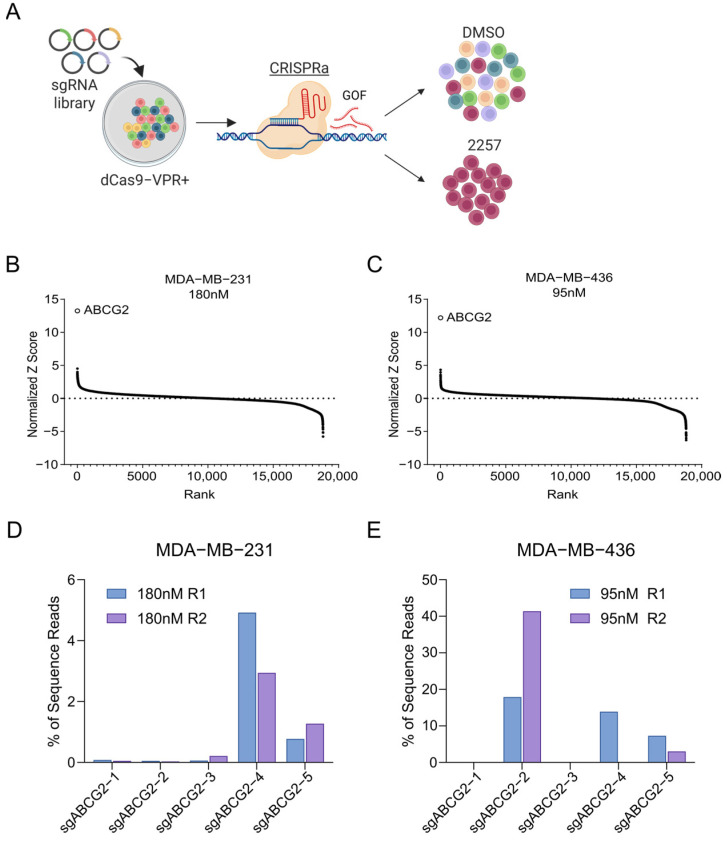
CRISPR activation screens identify ABCG2 as a putative 2257 resistance-conferring gene in TNBC cell lines. (**A**) Schematic indicating the design of genome-wide CRISPRa screens to discover genes whose GOF confers resistance to 2257 in TNBC models. sgRNAs induce expression of target genes in dCas9-VPR+ cells. (**B**,**C**) Visualization of screen hits identified using the Drug Z algorithm in MDA-MB-231 (**B**) and MDA-MB-436 (**C**) cells treated with an IC_95_ dose of 2257. Rank indicates the rank of individual genes assessed in the screen, and normalized Z-score represents a composite score indicating the relative abundance of all sgRNAs targeting specific genes in 2257-treated cells compared to cells collected at the time of screen onset. Positive Z-scores indicate sgRNAs that are enriched in 2257-treated cells. ABCG2 was the top hit identified in both cell lines. (**D**,**E**) sgRNAs targeting ABCG2 comprise a substantial fraction of total sgRNA sequencing reads in the MDA-MB-231 and MDA-MB-436 CRISPRa screens. The percentage of total sgRNA reads obtained for each 2257-treated replicate at the end of the screen is plotted for each sgRNA targeting ABCG2 in the hCRISPRa-v2 sgRNA library. sgRNA abundances at T0 are not shown because they were <0.1% of reads in both cell lines.

To discover cell-intrinsic mechanisms of resistance to 2257 conferred by gene GOF, we conducted pooled CRISPR activation (CRISPRa) screens in two commonly used TNBC cell lines: MDA-MB-231 and MDA-MB-436. Positive selection screens to detect sgRNAs enriched in the presence of lethal doses of 2257 were conducted to discover genes whose overexpression confers resistance to 2257-mediated TTK inhibition (Figure 1A) [14]. Briefly, sgRNA library-infected cells were subjected to culture in growth media supplemented with an IC_80_ or IC_95_ dose of 2257 or DMSO vehicle control [MDA-MB-231 150 nM and 180 nM; MDA-MB-436 80 nM and 95 nM] and passaged for 10 cell doublings (Figure S1C,D), at which point genomic DNA was harvested for targeted sgRNA sequencing. sgRNA abundance in 2257-treated cells at the endpoint was compared to that in library-transduced cells at screen onset to identify sgRNAs enriched in drug-treated conditions and the genes they target. Remarkably, ABCG2 was identified as the top hit in both cell lines at both 2257 doses (Figure 1B,C and Figure S1E,F). ABCG2 is an ATP-dependent ABC transporter with broad substrate specificity and a well-established mediator of multidrug resistance attributable to its drug efflux activity [16]. sgRNA targeting ABCG2 accounted for approximately 5% and 75% of all reads generated for individual treatment replicates in the MDA-MB-231 and MDA-MB-436 screens, respectively, indicating dominance of ABCG2 as a putative resistance mechanism (Figure 1D,E and Figure S1G,H). Complementing our CRISPRa screens, we also conducted a CRISPR/Cas9 LOF screen with a custom sgRNA library targeting “druggable” genes [17] to identify targets whose inactivation sensitized MDA-MB-231 cells to a sublethal dose of 2257 (50 nM) (Figure S2A). This screen also identified ABCG2 as the top hit, as sgRNA targeting ABCG2 were significantly depleted from the 2257-treated population (Figure S2B).

### 2.2. ABCG2 Expression Confers Resistance and Is a Putative Biomarker of Response to 2257 in Cancer Cell Lines

**Figure 2:**
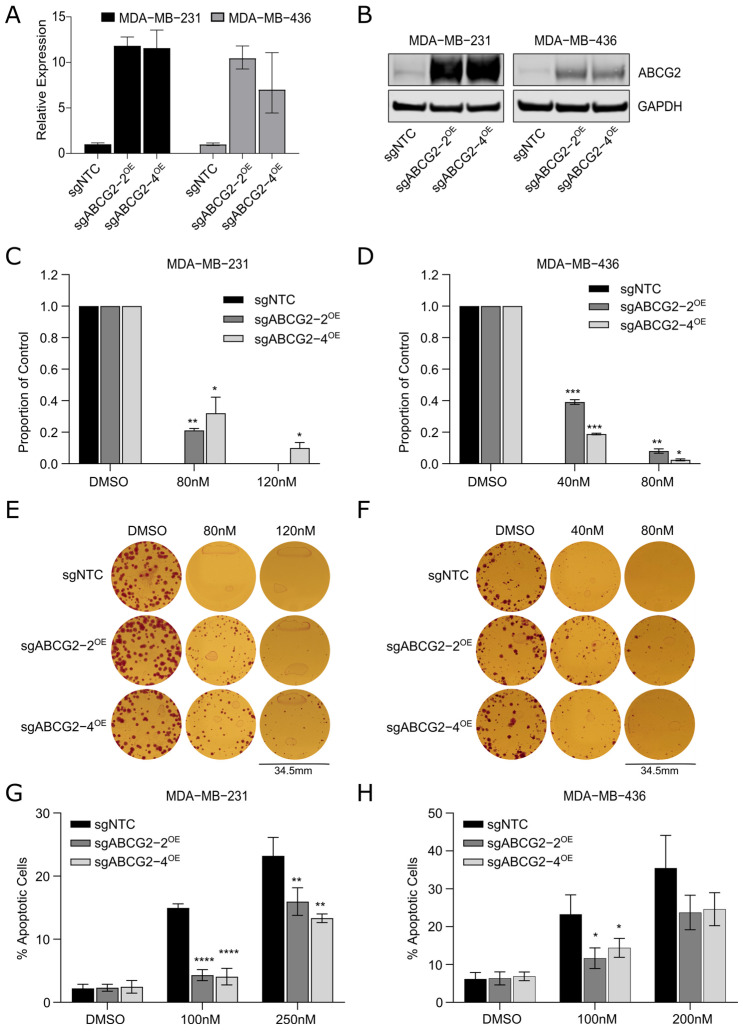
ABCG2 overexpression confers resistance to 2257. (**A**,**B**) Confirmation that MDA-MB-231 and MDA-MB-436 dCas9-VPR+ cells expressing sgABCG2-2^OE^ and sgABCG2-4^OE^ have elevated ABCG2 expression by qPCR (**A**) and Western blotting (**B**). Representative experiments are shown. (**C**,**D**) Cells overexpressing ABCG2 have increased capacity for clonogenic survival upon 2257 treatment. Cells were plated sparsely and treated with DMSO or 2257 at the doses indicated. After 10–14 days, colonies were counted. The proportion of surviving colonies for each derivative line is normalized to the number of colonies that grew in the DMSO-treated control. Error bars indicate the mean ± SD of 3 experimental replicates. (**E**,**F**) Representative images of colony survival in DMSO- and 2257-treated MDA-MB-231 (**E**) and MDA-MB-436 (**F**) cells. (**G**,**H**) ABCG2 overexpression suppresses apoptosis induced by 2257 treatment. Apoptosis was measured in cells treated with DMSO or the indicated doses of 2257 by flow cytometry to measure Annexin V-DAPI staining. Error bars indicate the mean ± SD of 4 biological replicates. * Two-tailed unpaired *t*-test, *p* < 0.05, ** *p* < 0.01, *** *p* < 0.001, **** *p* < 0.0001.

We next sought to functionally validate that ABCG2 drives resistance to 2257 in our TNBC models outside of the pooled CRISPR screen context. MDA-MB-231 and MDA-MB-436 dCas9-VPR+ cells were transduced with two distinct sgRNAs that were enriched in our screens (Figure S1G,H) and verified to induce ABCG2 expression (Figure 2A,B and Figure S3A–D). Clonogenic survival assays were used to evaluate the effect of ABCG2 overexpression (OE) on the viability of cells treated with 2257. The results indicated that cells overexpressing ABCG2 had significantly greater capacity to form colonies in the presence of 2257 at doses that severely restricted colony outgrowth in control cells (Figure 2C–F). ABCG2 overexpression also reduced the abundance of apoptotic cells induced by acute treatment with 2257 in both models (Figure 2G,H). These data provide evidence that inducing expression of ABCG2 confers resistance to 2257, validating ABCG2 as a hit from our CRISPRa screens. We next asked whether ABCG2 expression could be used to predict cell line response to 2257. To investigate this idea, we leveraged 2257 drug response data for over 600 cancer cell lines generated using the Broad Institute’s PRISM drug screening platform. Pharmacogenomic analyses to identify molecular features associated with cell line response revealed that mRNA expression of ABCG2 was positively correlated with resistance to 2257 (Figure S2C), evidenced by greater ABCG2 expression in cell lines with higher area under the curve values calculated from dose–response data. This correlation is consistent with data from our CRISPR screens, supporting the association between ABCG2 expression and resistance to 2257 and implicating ABCG2 as a putative biomarker for predicting 2257 response in cell models.

### 2.3. ABCG2 Likely Enhances Efflux of 2257 to Confer Resistance In Vitro

**Figure 3:**
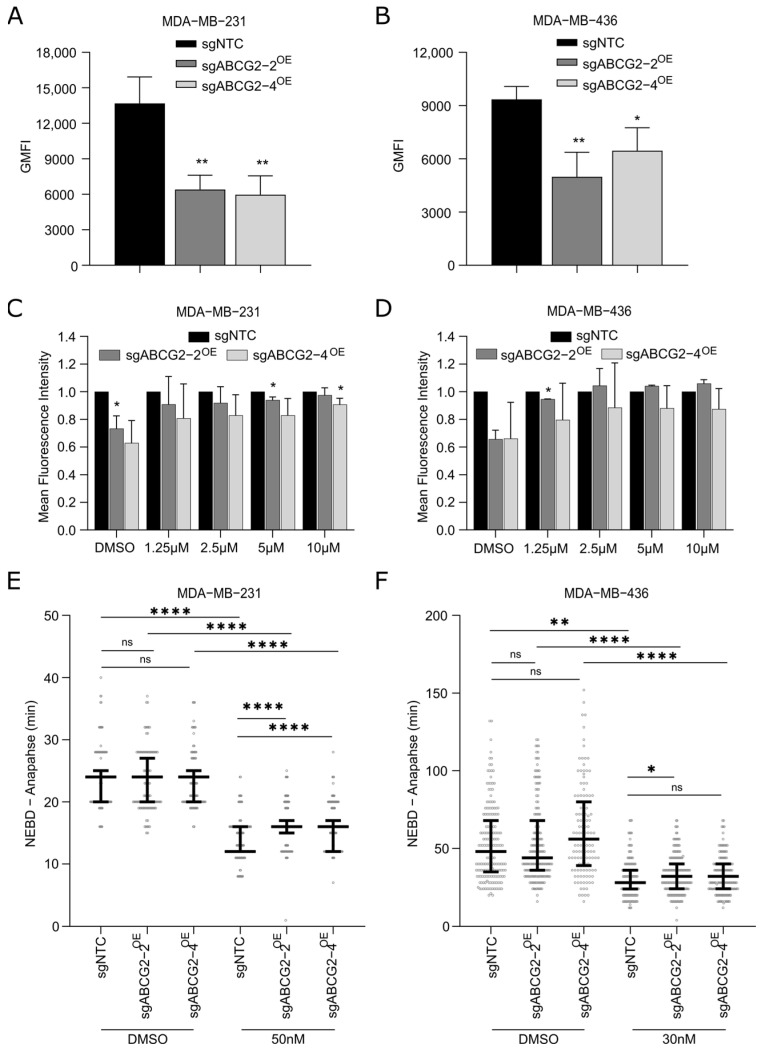
ABCG2 overexpression is associated with increased substrate efflux activity that is reversed by ABCG2 inhibition with FTC. (**A**,**B**) MDA-MB-231 and MDA-MB-436 cells overexpressing ABCG2 have reduced intracellular Hoechst fluorescence compared to controls. Cell lines were incubated with the established ABCG2 substrate, Hoechst 33342, and the geometric mean fluorescence intensity was quantified by flow cytometry. Error bars indicate the mean ± SD of 3 independent experiments. (**C**,**D**) ABCG2 inhibition in ABCG2-overexpressing cells rescues Hoechst fluorescence. Cells were treated with serial dilutions of the ABCG2 inhibitor, fumitremorgin C (FTC), and then incubated with Hoechst 33342. Cellular fluorescence levels were measured on a spectrophotometer, and values were normalized to levels observed in sgNTC cells. Error bars indicate the mean ± SD of 3 (MDA-MB-231) and 2 (MDA-MB-436) biological replicates. (**E**,**F**) ABCG2 overexpression diminishes 2257-mediated inhibition of the spindle assembly checkpoint (SAC). Live-cell, time-lapse microscopy was done on cells treated with DMSO or 2257 at the indicated doses and the fluorescent DNA stain, siRDNA, to monitor the time between nuclear envelope breakdown (NEBD) and anaphase as a readout of SAC activity. Results show 2257 abrogates the delay in mitotic progression caused by SAC activation, thereby shortening NEBD–anaphase time in sgNTC cells. ABCG2 overexpression increases NEBD–anaphase time in 2257-treated cells, diminishing 2257’s effects on SAC inhibition. Data shown are pooled from two independent experiments for MDA-MB-231 and 4 independent experiments for MDA-MB-436. At least 120 mitotic cells were counted per condition. Error bars indicate the median with interquartile range. * Two-tailed unpaired *t*-test, *p* < 0.05, ** *p* < 0.01, **** *p* < 0.0001, ns = not significant.

Given that ABCG2 functions as a drug efflux pump and is known to mediate resistance to a number of anti-cancer drugs [16], we suspected 2257 could be a substrate of ABCG2. Thus, we sought to confirm that overexpression of ABCG2 leads to enhanced ABCG2 efflux activity. Upon treating cells with Hoechst, a fluorescent ABCG2 substrate [18], we found that TNBC cells with ABCG2 overexpression had significantly reduced intracellular fluorescence compared to controls, consistent with active efflux of Hoechst by ABCG2 (Figure 3A,B). To confirm the involvement of ABCG2 activity in mediating this phenotype, we tested two inhibitors of ABCG2 in MDA-MB-231 cells in which ABCG2 expression reduced intracellular Hoechst by over 2-fold (Figure 3A): the mycotoxin Fumitremorgin C (FTC), a well-established inhibitor of ABCG2 [19], and Febuxostat, a more recently described ABCG2 inhibitor [20]. While FTC did not have an effect on intracellular Hoechst in sgNTC control cells with low endogenous levels of ABCG2, a dose-dependent increase in intracellular Hoechst fluorescence was observed in FTC-treated cells with ABCG2 overexpression (Figure 3C,D and Figure S4A,B). We did not observe a rescue in intracellular Hoechst levels in ABCG2-overexpressing MDA-MB-231 cells upon treatment with up to 10 µM of Febuxostat, suggesting it cannot effectively inhibit ABCG2 in these cells at the doses tested (Figure S4C,D). These data illustrate that FTC can reverse the reduction in intracellular Hoechst fluorescence driven by ABCG2 overexpression, implicating ABCG2 as a mediator of Hoechst efflux.

To test whether ABCG2 confers resistance to 2257 by minimizing its intracellular accumulation, we examined the biological effects of 2257 on cells with and without ABCG2 overexpression. Since TTK activates the SAC to delay anaphase in mitotic cells, 2257-mediated TTK inhibition reduces the amount of time it takes for dividing cells to transit mitosis. Our previous characterization of 2257 in TNBC models showed that TTK inhibition induces cytotoxicity by accelerating the duration of mitosis (i.e., nuclear envelope breakdown (NEBD) to anaphase time), which causes lethal chromosome segregation errors in dividing cells [14]. As such, we posited that 2257-treated cells overexpressing ABCG2 would exhibit longer NEBD-to-anaphase times than control cells with basal levels if ABCG2 were actively exporting 2257 out of the cells. Time-lapse live-cell microscopy revealed no differences in mitotic duration in DMSO-treated cells, but NEBD-to-anaphase time was significantly longer in 2257-treated MDA-MB-231 cells with ABCG2 overexpression compared to sgNTC controls (Figure 3E), consistent with our hypothesis. Although not significant for both lines overexpressing ABCG2, the same trend was observed in MDA-MB-436 (Figure 3F). These results suggest ABCG2 confers resistance to 2257 by efflux of 2257 to diminish its effects on the SAC and resulting cytotoxicity.

### 2.4. Inhibition of ABCG2 Sensitizes Breast Cancer Cell Lines to 2257

**Figure 4:**
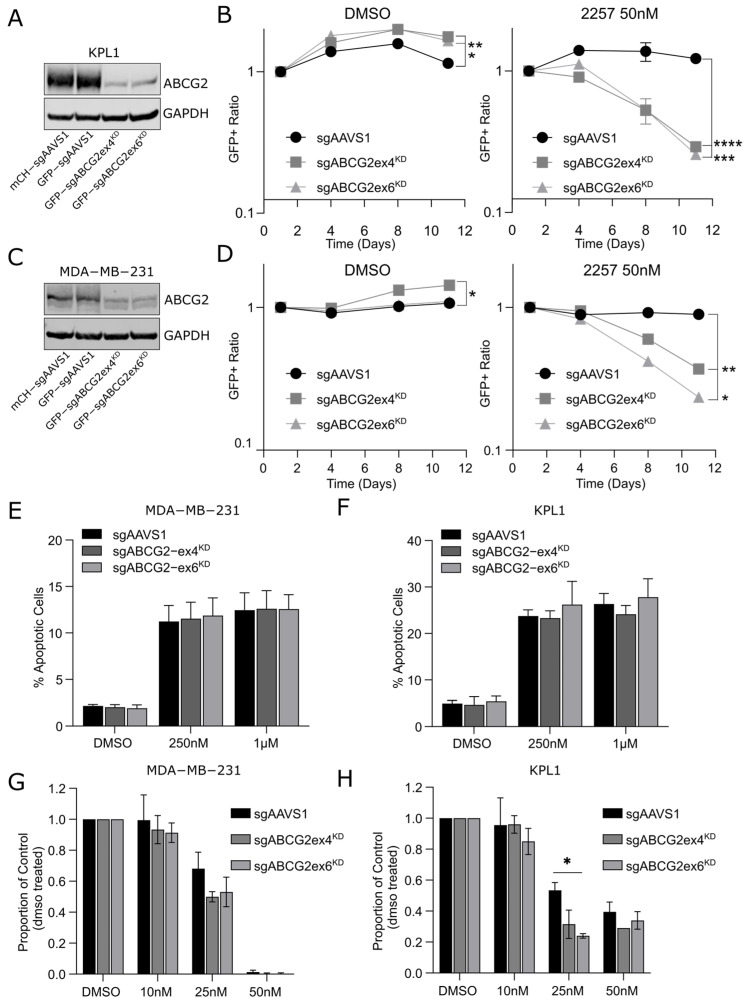
Knockdown of ABCG2 potentiates 2257 activity in breast cancer cells. (**A**,**C**) Western blot confirmation of CRISPR-mediated ABCG2 knockdown in KPL1 (**A**) and MDA-MB-231 (**C**) breast cancer cells. (**B**,**D**) Multi-color competition assays to assess the relative fitness of cells with endogenous ABCG2 expression (GFP+ and mCh+ sgAAVS1) and ABCG2 KD (GFP+sgABCG2ex4^KD^ and GFP+sgABCG2ex6^KD^). Briefly, GFP+ cell lines were mixed in equal proportions with mCh+sgAAVS1 cells, and the ratio of GFP+ to mCh+ cells treated with DMSO or 50 nM 2257 was monitored over multiple passages using flow cytometry. The mixture of mCh+sgAAVS1 and GFP+sgAAVS1 cells serves as a negative control. The decline in GFP+ ratio for cell mixtures competing GFP+sgABCG2ex4^KD^ and GFP+sgABCG2ex6^KD^ cells against mCh+sgAAVS1 cells in 2257-treated conditions indicates that cells with ABCG2 KD have a fitness disadvantage in the presence of 2257. Data shown are representative of 3 independent experiments. (**E**,**F**) Knockdown of endogenous ABCG2 expression does not potentiate apoptosis induced by acute 2257 treatment. Cells with ABCG2 KD were treated with DMSO or the indicated doses of 2257 for 72 h, and apoptosis was measured using Annexin V-DAPI staining. Error bars indicate the mean ± SD of 3 biological replicates. (**G**,**H**) Reducing basal ABCG2 expression may impair the clonogenic survival of MDA-MB-231 and KPL1 cells treated with low-dose 2257. Colonies were counted after 10–14 days of treatment. Error bars indicate the mean ± SD of 3 (MDA-MB-231) and 2 (KPL1) biological replicates. * *p* < 0.05, ** *p* < 0.01, *** *p* < 0.001, **** *p* < 0.0001.

In addition to our CRISPRa screens identifying ABCG2 as a hit, CRISPR dropout screens in MDA-MB-231 cells revealed ABCG2 LOF as a potential 2257 sensitization mechanism (Figure S2B). To validate that genetically mediated inhibition of ABCG2 sensitizes cells to 2257, we conducted multicolor competition assays. We selected two breast cancer lines with relatively high endogenous ABCG2 expression, MDA-MB-231 and KPL1 (luminal subtype), to compare the relative fitness of 2257-treated cells with and without CRISPR-mediated ABCG2 knockdown (KD). We engineered Cas9+GFP+ cells with ABCG2 KD using two different sgRNAs (sgABCG2ex4 and sgABCG2ex6), as well as Cas9+mCh+ and Cas9+GFP+ cells expressing an sgRNA targeting the safe harbor locus, AAVS1 (sgAAVS1), as negative controls (Figure 4A,C). Then, we mixed equal proportions of each GFP+ line with the mCh+sgAAVS1 line, treated the cells with 2257 or DMSO, and monitored the ratio of GFP+ to mCh+ cells over several passages. These assays indicated that ABCG2 KD conferred a slight fitness advantage in DMSO-treated MDA-MB-231 and KPL1 cells (Figure 4B,D). Conversely, cells with ABCG2 KD were significantly depleted from cell mixtures treated with 2257, evident by significant decreases in the ratio of GFP:mCh+ cells over time (Figure 4B,D), validating ABCG2 as a hit in our 2257 sensitization screens. We observed no differences in apoptosis after acute 2257 treatment (Figure 4E,F), consistent with depletion of cells with ABCG2 KD being evident at later time points in our competition assays. In chronic 2257 treatment experiments, we found non-significant trends towards impaired clonogenic survival in cells with ABCG2 KD compared to sgAAVS1 controls (Figure 4G,H). Collectively, these studies suggest that genetic inactivation of ABCG2 sensitizes breast cancer cells to 2257.

**Figure 5:**
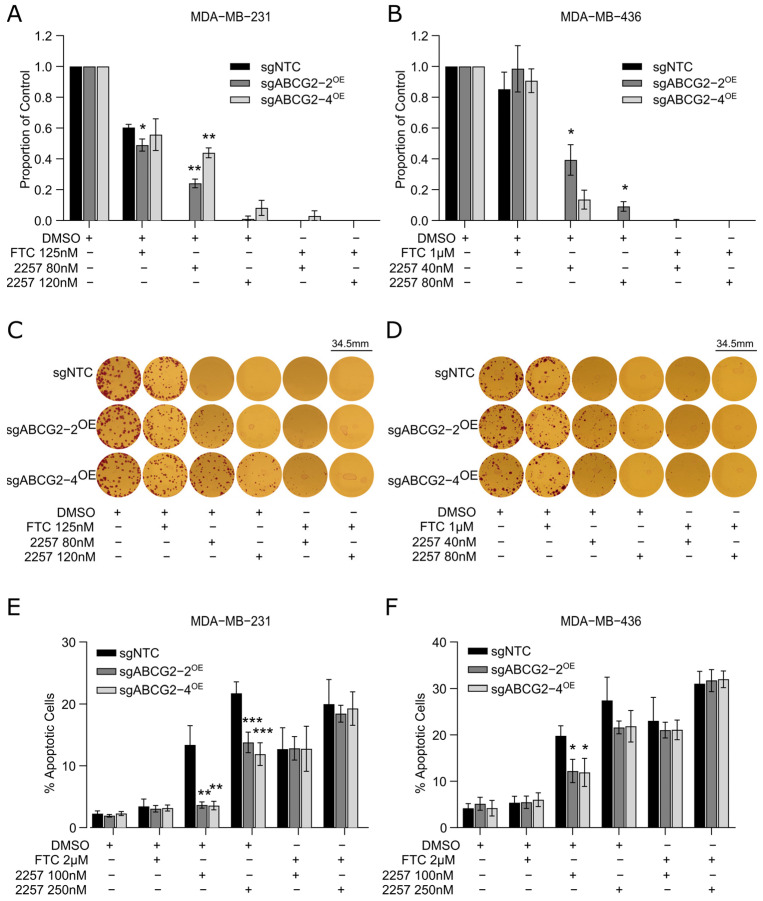
Pharmacologic inhibition of ABCG2 overcomes ABCG2-mediated resistance to 2257. (**A**,**B**) ABCG2 inhibition sensitizes MDA-MB-231 and MDA-MB-436 cells overexpressing ABCG2 to 2257. Clonogenic survival assays were conducted with the indicated 2257 and FTC treatment conditions. Error bars indicate the mean ± SD of 3 biological replicates. (**C**,**D**) Representative images of colony survival in MDA-MB-231 (**C**) and MDA-MB-436 (**D**) cells treated with and without 2257 and FTC. (**E**,**F**) FTC treatment rescues the induction of apoptosis by 2257 in cells overexpressing ABCG2. Apoptosis was measured using flow cytometry for Annexin V-DAPI in treated cells. Error bars indicate the mean ± SD of 4 (MDA-MB-231) and 3 (MDA-MB-436) biological replicates. * *p* < 0.05, ** *p* < 0.01, *** *p* < 0.001.

To assess the potential for pharmacologic ABCG2 inhibition to reverse resistance and rescue 2257 sensitivity, TNBC cells overexpressing ABCG2 were treated with 2257 ± FTC, and clonogenic survival and apoptosis were measured as readouts of sensitivity. We found that FTC sensitized ABCG2-overexpressing cells to 2257, evident by near-complete inhibition of clonogenic survival in MDA-MB-231 and MDA-MB-436 cells (Figure 5A–D). Furthermore, FTC restored apoptosis induced by 2257 in cells with ABCG2 overexpression to levels similar to those observed in control cells with endogenous ABCG2 expression in both TNBC models (Figure 5E,F). These findings indicate that ABCG2 inhibition sensitizes TNBC cells to 2257 to overcome resistance, suggesting ABCG2-targeted drugs could be used to potentiate response to 2257.

### 2.5. ABCG2 Overexpression Does Not Confer Resistance to 2257 In Vivo in MDA-MB-231

**Figure 6:**
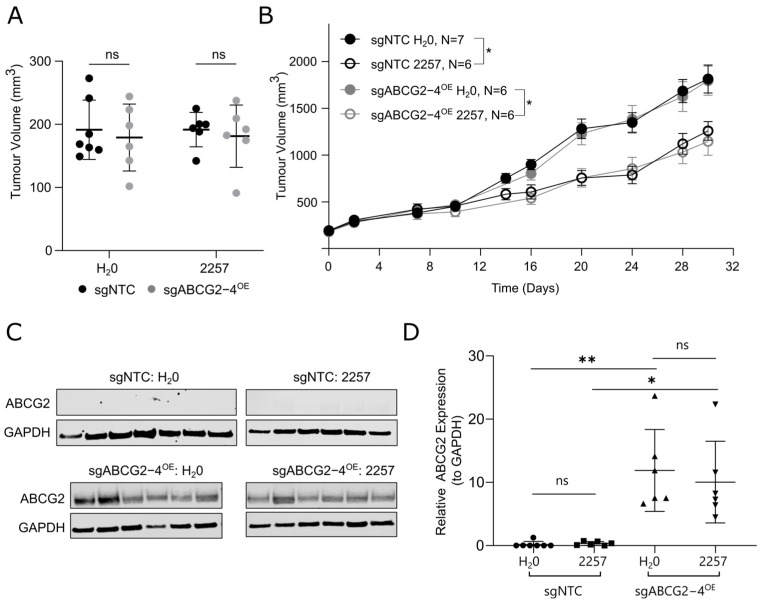
Overexpression of ABCG2 in MDA-MB-231 xenografts does not confer resistance to 2257 in vivo. MDA-MB-231-dCas9-VPR+ cell lines with and without ABCG2 overexpression were implanted into the mammary fat pads of female SCID mice and treated daily with H_2_O or 2257 (6 mg/kg) by oral gavage. Mice were euthanized when the average tumor volume for control-treated animals reached ~1500–2000 mm^3^. (**A**) Volumes of tumors with or without ABCG2 overexpression at the time of randomization into treatment groups. (**B**) Growth curves for sgNTC versus sgABCG2-4^OE^ tumors. The number of mice in each treatment arm is indicated. Error bars indicate the mean ± SEM for each treatment group. Asterisks indicate significance for differences in tumor volumes at endpoint only. (**C**) Confirmation of ABCG2 overexpression in tumors at the experimental endpoint. Protein lysates were prepared from tumor tissues resected following euthanasia, and ABCG2 was detected by Western blots. (**D**) Quantification of ABCG2 expression in tumors resected at endpoint from each treatment arm. ABCG2 expression levels were normalized to GAPDH. * *p* < 0.05, ** *p* < 0.01, ns = not significant.

Lastly, we investigated the effects of ABCG2 overexpression on breast cancer tumor response to 2257 in vivo. Given the robust resistance-conferring phenotype we observed in the MDA-MB-231 model in vitro, we engrafted MDA-MB-231 cells with ABCG2 overexpression (sgABCG2-4^OE^) and matched controls (sgNTC) into SCID mice. We hypothesized that tumors with ABCG2 overexpression would be relatively resistant to 2257, consistent with our in vitro observations. Tumor-bearing mice were randomized into groups balanced for tumor size and treated with 6 mg/kg/day 2257 or H_2_O as a control (Figure 6A). Consistent with the in vitro sensitivity observed, 2257 elicited anti-tumor effects in MDA-MB-231 sgNTC control tumors, with treated tumors exhibiting a 31% reduction in tumor volume at endpoint (Figure 6B). Unexpectedly, however, tumors overexpressing ABCG2 were equally sensitive to 2257 as sgNTC controls (36% tumor growth inhibition; Figure 6B). To determine whether ABCG2 overexpression was maintained in vivo, we harvested tumors from mice at endpoint to measure ABCG2 levels. Immunoblotting confirmed that ABCG2 was highly overexpressed in sgABCG2 compared to sgNTC controls (Figure 6C,D), indicating that lack of ABCG2 expression could not explain the lack of tumor resistance to 2257 in mice. Thus, despite our studies providing strong evidence that ABCG2 confers resistance to 2257 in vitro, ABCG2 had no appreciable effect on the 2257 response at the dose tested in vivo.

## 3. Discussion

Understanding tumor response to therapy is a critical component of preclinical drug development, as discovering mechanisms governing sensitivity and resistance can reveal biomarkers to guide patient selection for therapy and can influence the design of clinical trials. For instance, identifying proteins that confer drug resistance can inform the development of clinical assays for testing their expression in tumor biopsies to avoid treating patients with drugs unlikely to be effective. In this study, we used genetic screens to discover genes whose activation confers breast cancer cell resistance to the TTK inhibitor, CFI-402257, and identified ABCG2 as the top resistance-conferring gene. Our findings provided robust evidence implicating ABCG2 as a mediator of response to 2257 in cell models, which we validated using genetic and pharmacologic approaches. Unexpectedly, however, ABCG2 did not influence the response of TNBC xenografts to active doses of 2257 in mice.

Drug efflux is one of the most common mechanisms underlying multidrug resistance (MDR) in cancer cells [16], and ATP-binding cassette transporter G2, ABCG2, is one of the best characterized MDR proteins. Powered by ATP hydrolysis, ABCG2 transports endogenous compounds, including hormones and nutrients, to maintain organismal homeostasis and also pumps xenobiotics like drugs out of cells to limit their cytotoxic effects [16,21,22]. For example, ABCG2 expression at the blood–brain barrier and in the placenta functions to protect the brain and fetus from harmful endo- and exotoxins [22,23]. Well-established drug substrates of ABCG2 include the anthracycline doxorubicin, the antimetabolite fluorouracil, the EGFR tyrosine kinase inhibitor gefitinib, and the estrogen antagonist tamoxifen [22,23]. Although we did not conduct experiments to directly assess whether 2257 is a physical substrate of ABCG2, our live-cell mitotic imaging supported this idea. We previously showed that 2257-mediated inhibition of the SAC accelerates cell transit through mitosis and that prolonging mitotic duration to counter this effect confers TNBC resistance to 2257-mediated SAC inhibition [14]. In this study, we found that 2257-treated cells overexpressing ABCG2 spent longer in mitosis than controls with basal expression levels, concordant with a lower intracellular concentration of 2257 and diminished inhibition of the SAC. Consistent with reduced levels of 2257 within cells, we also observed diminished cytotoxic effects, evident by enhanced clonogenic survival and reduced apoptosis in 2257-treated TNBC cells with ABCG2 overexpression. These findings suggest that 2257 is indeed a substrate of ABCG2’s drug efflux pump activity in in vitro conditions at pharmacologically active concentrations.

Our results indicated that genetically mediated knockdown of endogenous ABCG2 had no effect on apoptosis induced by 2257 in acutely treated breast cancer cells, but we observed greater sensitivity to 2257 in clonogenic survival and competitive fitness assays in which cells with ABCG2 knockdown were treated with 2257 for longer periods of time. We suspect this could indicate that reducing ABCG2 expression in cells with low endogenous expression is not biologically relevant, or the change in ABCG2 knockdown that we achieved is insufficient to influence drug response. This could also suggest that ABCG2 becomes functionally relevant in the context of chronic treatment, leading us to speculate that ABCG2 may be upregulated to influence drug sensitivity over time. In agreement with this, the 2257 resistance phenotypes we observed in TNBC cells were achieved by inducing high levels of ABCG2 expression using CRISPRa methods. Several independent studies have reported upregulation of ABCG2 in cancer cell lines with acquired resistance to different chemotherapies [24,25]. In addition, evaluation of ABCG2 expression in treatment-naive tumors has implicated ABCG2 in mediating innate chemotherapy resistance, as multiple reports have identified higher levels of ABCG2 correlating with worse therapeutic response and poor clinical outcomes in different tumor types [22,23,26,27,28,29]. However, studies reporting associations between primary tumor ABCG2 expression and patient prognosis have shown conflicting results, indicating that further investigation is required to resolve its biomarker potential and define specific patient populations in which ABCG2 expression could serve as a predictor of drug response [23,26]. Nevertheless, the work of several studies nominates ABCG2 as a putative biomarker of resistance to drugs that are known ABCG2 substrates. If ABCG2 expression could be assessed in tumors from patients who experience recurrence after chemotherapy, this information could be leveraged to choose effective next lines of treatment, avoiding the use of ABCG2 substrate drugs whose efficacy would be limited by their efflux activity.

In contrast to the association we identified between ABCG2 expression and 2257 response in cancer cell lines, our tumor treatment studies did not confirm a role for ABCG2 expression in mediating TNBC response to 2257 in vivo. Despite robust evidence from genetic screens indicating ABCG2 confers resistance to 2257 in cell models, as well as functional studies suggesting 2257 is a substrate of its drug efflux activity, overexpression of ABCG2 did not confer resistance to 2257 administered at a moderately active dose and schedule in MDA-MB-231 tumor xenografts. This finding was unanticipated given that 2257 suppressed the growth of parental MDA-MB-231 tumors and ABCG2 overexpression strongly promoted resistance in cultured MDA-MB-231 cells. It is possible that the pharmacokinetics of 2257 and ABCG2 substrate selectivity in vitro and in vivo could underlie this observation. In our in vitro studies, we observed ABCG2-mediated resistance to 2257 at doses of 80 nM to 250 nM in TNBC cells. However, plasma concentrations of 2257-treated mice were found to be much greater, as repeat dosing of mice with 6.5 mg/kg daily yielded maximum plasma concentrations over 2 µM [9]. Thus, in vivo dosing may increase the ratio of 2257 to ABCG2 protein to a level that exceeds the ability of ABCG2 to effectively efflux 2257 from cells in vivo. In line with this idea, erlotinib was described to have concentration-dependent effects on ABCG2 transport activity, being transported out of cells at low concentrations and inhibiting ABCG2-mediated transport at high concentrations [30].

Differences in growth conditions experienced by cancer cells in culture and in mice could also limit ABCG2’s ability to confer resistance to 2257 in xenograft models. While cancer cell killing is dependent on cell-intrinsic drug effects in vitro, innate tumor immunity can also contribute to the anti-tumor effects of drugs that elicit immunogenic cell death in immunocompromised animals in vivo (e.g., SCID mice). We previously reported that 2257 treatment induces cytosolic DNA that activates the stimulator of interferon genes (STING) pathway in cancer cells, driving the expression and secretion of cytokines that recruit immune cells [13]. Engagement of the innate immune system through this mechanism could diminish the influence of ABCG2 on 2257 resistance in xenograft models. The doubling time of cancer cells grown in vivo is often slower than that in vitro, and anti-mitotic drugs can have less efficacy in tumors with low mitotic indices [31]. The differing proliferation rate may in part explain why 2257 dramatically reduced cancer cell populations in our CRISPR screens and only slowed tumor growth in vivo. ABCG2 overexpression may have enabled acute survival of rapidly proliferating cancer cells during the bottleneck induced by 2257 treatment in our screens, but 2257 failed to elicit enough cell death to abrogate tumor growth and induce tumor regression in vivo. The lack of this bottleneck effect in tumor xenografts may have diminished the relevance of ABCG2-mediated drug resistance in vivo. It is also possible that the selective pressure imposed by prolonged treatment with high-dose 2257 induced secondary mechanisms of resistance in our screened populations. These mechanisms, independent of CRISPR-mediated gene perturbation, could confer a more robust resistance phenotype that supersedes the effect of ABCG2, but they may not have had sufficient time to develop in response to 2257 treatment of tumors with low mitotic indices in mice.

Characteristics specific to the tumor microenvironment, including pH, ATP, cofactor and metabolite availability, presence of drug-binding proteins like albumin, abundance of other competing substrates, and activity of drug-metabolizing enzymes, may also influence ABCG2-mediated resistance in vivo by modifying drug metabolism, binding, and efflux by ABCG2 [16,32,33,34,35,36]. While sulfation, glucuronidation, and glutathione conjugation of drugs by phase II metabolizing enzymes often enhance drug binding to ABC transporters, efflux, and removal from cells, contributing to drug resistance [37,38]. These and other chemical modifications can also reduce the affinity of drugs for different efflux pumps [39,40]. This scenario is exemplified by the thrombin inhibitor and prodrug, dabigatran etexilate, as well as buprenorphine. While dabigatran etexilate is a substrate of the ABC transporter p-glycoprotein (P-gp), its metabolized active form, dabigatran, is not [39]. Similarly, norbuprenorphine, produced by phase II enzyme-mediated metabolism of buprenorphine, can be effectively effluxed by P-gp, unlike its glucuronidated form, buprenorphine glucuronide [40]. Thus, differences in drug disposition in vitro and in vivo could explain the failure of ABCG2 to confer breast tumor resistance to 2257 in mice.

We acknowledge that our MDA-MB-231 xenograft model of TNBC tumor growth and 2257 treatment does not fully recapitulate treatment in patients, since the drug concentrations achieved, dosing schedules, and time course for therapy differ. These factors are likely to influence tumor response to 2257. As such, our in vivo system may be limited in its ability to faithfully model the clinical course of treatment, and therefore, its ability to accurately define the biological relevance of ABCG2 in mediating TNBC resistance to 2257. Nevertheless, our finding that ABCG2 may have a different ability to confer resistance to 2257 in vitro and in vivo highlights the need to carefully interpret the findings of functional screens designed to identify molecular determinants of drug response in cancer cell lines. CRISPR screens can identify diverse molecular mechanisms underlying drug response, including those directly related to the mechanism of action of a drug. For example, we previously found that resistance to 2257-mediated SAC inhibition is conferred by genetic perturbations that prolong mitotic duration or delay mitotic exit [14]. However, screens may also reveal mechanisms related to the pharmacokinetic and pharmacodynamic properties of a drug, including metabolism and transport by enzymes that alter its pharmacologic effects. These factors can influence drug response but may have differential relevance in cell versus animal systems. This emphasizes the importance of validating the physiological relevance of hits discovered from in vitro CRISPR screens in animal models, despite their imperfections, to better understand their clinical significance and potential utility as biomarkers or therapeutic targets.

Additional insights brought to light by our work warrant careful consideration by researchers interpreting the results of GOF studies and designing sgRNA libraries for CRISPRa screens. Some screen “hits” may be driven by inducing target genes to supraphysiological expression levels that are not biologically relevant or achievable in physiological or pathological contexts, yielding false positive mechanisms in CRISPRa screens. In contrast, GOF screens may also yield false negative results for genes that are not effectively induced by sgRNA in genome-wide CRISPRa libraries. Unlike for ABCG2, we have been unable to induce genes of interest for other projects using sgRNA from published libraries, suggesting that the principles for CRISPRa sgRNA design could benefit from further optimization to yield a greater proportion of guides that effectively induce gene expression. Lastly, sgRNA targeting ABCG2 in our GOF screens accounted for up to 75% of the total sgRNA reads in some of our screen replicates. This level of enrichment could reduce screen sensitivity by limiting reads available to detect enrichment of sgRNA targeting other genes. This is exemplified by the fact that we detected 11 putative hits besides ABCG2 in our MDA-MB-231 screens, which had much lower enrichment of ABCG2 sgRNA, but only two other hits in MDA-MB-436, and removing sgRNA targeting ABCG2 from the analysis did not change the hits identified. Thus, determining whether drugs of interest are substrates for MDR proteins or influenced by drug-metabolizing enzymes is a worthwhile endeavor prior to conducting screens to avoid detection of genes encoding such proteins as prominent screen hits. This issue could be mitigated by removing sgRNA targeting such genes from genome-wide sgRNA libraries to prioritize identification of drug response mechanisms more directly related to their mechanisms of action rather than their pharmacokinetic and pharmacodynamic properties.

## 4. Materials and Methods

Cell lines and derivatives. MDA-MB-231, MDA-MB-436, and KPL-1 breast cancer lines were generous gifts from Dr. Benjamin Neel and were maintained in DMEM media supplemented with 2 mM glutamine, 100 U/mL of penicillin–streptomycin, and 10% fetal bovine serum (FBS). Models were engineered for CRISPR/Cas GOF and LOF screens by lentiviral transduction with *S. pyogenes* dCas9-VPR-mCherry (Addgene, Watertown, MA, USA, #154193) or Cas9 Blast (Addgene #52962). Cells with stable dCas9-VPR and Cas9 expression were selected by flow sorting mCherry+ cells and treatment with blasticidin (4 μg/mL), respectively. Derivative lines were subsequently sub-cultured to isolate clones with high efficiencies of gene induction (Figure S1A,B) and gene editing (previously reported [14]). Cell line identities were confirmed by short tandem repeat testing and were also regularly tested to confirm mycoplasma negativity. Lentivirus was produced as previously described [14].

CRISPR GOF screens. The Weissman genome-wide CRISPR activation (CRISPRa) library containing 104,540 sgRNAs targeting 19,915 human genes was obtained from Addgene (hCRISPRa-v2; #83978) [41]. Positive selection enrichment screens were conducted as previously described to discover genes whose activation confers resistance to 2257 [14]. Clonal MDA-MB-231 and MDA-MB-436 dCas9-VPR+ cells were infected with lentivirus encoding the hCRISPRa-v2 sgRNA library at an MOI of 0.4. Cells were selected with puromycin for 3 days and expanded on puromycin for an additional 8 days to obtain enough library-transduced cells for two biological replicates of each screen condition. At the time of screen initiation, cells were divided into pools of 30 million cells (~300× library coverage) for each experimental replicate, and the remaining cells were collected to measure baseline sgRNA representation (T_0_). Treatments consisted of two lethal doses of 2257 spanning the IC_80_-IC_95_ for each cell line (MDA-MB-231 at 150 nM and 180 nM; MDA-MB-436 at 80 nM and 95 nM). Cells were maintained in media +/− 2257 and counted at each passage, and screens were terminated after 10 cell doublings (Figure S1C,D). Genomic DNA (gDNA) was extracted from each screen population and T_0_ sample and subjected to targeted sgRNA sequencing at the Princess Margaret Genome Centre. DrugZ (https://github.com/hart-lab/drugz; accessed on 4 December 2019) was used to analyze sgRNA abundance across cell populations to identify sgRNAs significantly enriched in 2257-treated compared to T_0_ cells [42]. Genes were considered hits using a DrugZ FDR threshold of q < 0.25. We also removed sgRNA targeting ABCG2 from the raw sgRNA input data and re-analyzed it with DrugZ using the same settings and FDR threshold of q < 0.25 to determine whether removing ABCG2 sgRNAs modified the hit lists.

CRISPR LOF screen. A negative selection depletion screen was performed in the MDA-MB-231 model using a custom CRISPR knockout (KO) library containing 13,243 sgRNAs targeting 3319 unique genes classified as “druggable,” as previously reported [17] (Figure S2). Library-encoding lentivirus was introduced into clonal MDA-MB-231-Cas9+ cells at an MOI of 0.45, and transduced cells were selected for 3 days with puromycin (2 μg/mL). Immediately after selection was complete, cells were pooled and split into DMSO or 2257 (50 nM) treatment arms in triplicate, and cell pellets were collected for the T_0_ sgRNA readout. Infections were scaled to ensure enough library-infected cells were recovered post-selection to initiate screens with 400× library coverage for each experimental replicate. Cells were maintained on treatment and counted at each passage, and screens were terminated after 15 cell doublings. gDNA was subjected to targeted sgRNA sequencing at the Princess Margaret Genome Centre, and DrugZ was used as above to discover sgRNAs significantly depleted in 2257-treated conditions relative to DMSO-treated conditions [42]. Genes with a DrugZ FDR < 0.25 were considered screen hits.

Broad PRISM Screening. The Broad’s PRISM Lab was contracted for pharmacogenomic profiling experiments to discover molecular biomarkers of response to 2257. Briefly, 770 barcoded cancer cell lines were treated with 8 × 4-fold serial dilutions of 2257, with a max dose of 2.5 µM, in a multiplexed system. Viability for each drug dose after 5 days was digitally quantified using next-generation sequencing to measure the relative barcode abundance between DMSO- and 2257-treated conditions. In this manner, cell line sensitivity to each dose of 2257 was determined, and metrics including Area Under the Curve (AUC) were correlated with molecular features using univariate and multivariate models to identify biomarkers associated with response to 2257. ABCG2 mRNA expression was extracted for 590 cell lines with processed data available from the Cancer Cell Line Encyclopedia database and plotted versus PRISM-calculated AUCs to visualize the correlation between ABCG2 expression and 2257 sensitivity.

ABCG2 Validation Studies. For CRISPRa experiments, individual sgRNAs targeting ABCG2 or a non-targeting control (NTC; Table S1) were cloned into pRC0162 (pSB700-puro), a generous gift from Dr. Raj Chari. dCas9-VPR+ cells were infected with lentivirus encoding each construct for functional studies. Five ABCG2-targeting sgRNAs from the hCRISPRa-v2 library were tested (Figure S3), and two that robustly and specifically induced ABCG2 overexpression (OE) were selected for validation studies (#2, sgABCG2-2^OE^ and #4, sgABCG2-4^OE^). For CRISPR knockdown (KD) studies, independent sgRNAs targeting exons 4 and 6 of the ABCG2 gene (sgABCG2ex4^KD^ and sgABCG2ex6^KD^; Table S1) were cloned into pLenti-Guide-GFP (Addgene #185473). Lentiviruses encoding these constructs, as well as pLenti-Guide-GFP and pLenti-Guide-mCh (Addgene #185474) constructs containing an sgRNA targeting AAVS1 as a negative control, were produced and used to infect Cas9+ cells to generate cells expressing mCh+sgAAVS1, GFP+sgAAVS1, GFP+sgABCG2ex4^KD^, and GFP+sgABCG2ex6^KD^. pLenti-Guide vectors were generously provided by Dr. Daniel Durocher. ABCG2 KD and OE were confirmed with RT-qPCR using SYBR-green reagent and/or Western blotting using anti-ABCG2 (Cell Signaling #42078 at 1:500 dilution, Danvers, MA, USA) and anti-GAPDH (Cell Signaling #2118 at 1:1000 dilution) antibodies. qPCR primer sequences are provided in Table S1.

Cell Viability Assays. For clonogenic survival assays, 400–1000 cells were seeded in 6-well plates in triplicate and treated with DMSO or 2257 the following day. After 10–14 days, colonies were fixed with 10% TCA, stained with SRB, and manually counted. Relative clonogenic survival was calculated by dividing the number of colonies in 2257-treated wells by the number of colonies in DMSO-treated wells and comparing these proportions across genotypes. Drug-induced apoptosis was measured using Annexin V-DAPI staining of cells treated with DMSO or 2257. Cells were collected for staining and flow cytometry after 72 h (KPL1, MDA-MB-231) or 5 days (MDA-MB-436) of treatment, and those positive for DAPI, Annexin V, or both were classified as apoptotic.

ABCG2 Activity Assays. Hoechst 33342 fluorescence was assessed in cells as a measure of ABCG2 efflux pump activity since Hoechst 33342 is an established ABCG2 substrate [18]. For flow cytometry readouts, 1 million cells were stained with media containing 5 μg/mL Hoechst 33342 for 15 min at 37 °C and subsequently analyzed on a BD Fortessa cytometer to measure fluorescence. For microplate readouts of cells treated with the ABCG2 inhibitors Fumitremorgin C (Sigma-Aldrich, St. Louis, MO, USA) and Febuxostat (MedChemExpress, Monmouth Junction, NJ, USA) [19,20,21], cells were seeded in 96-well plates. The next day, cells were treated with media containing serial dilutions of the inhibitors for 30–60 min at 37 °C and subsequently stained with Hoechst 33342 for 15 min at 37 °C. Hoechst fluorescence was measured on a SpectraMax multi-mode microplate reader (Molecular Devices, San Jose, CA, USA) with excitation at 350 nm and emission at 461 nm.

Live-Cell Microscopy. Cells were plated in Nunc Lab-Tek II chamber slides in phenol-red-free DMEM containing 167 nM siRDNA (Cytoskeleton, Denver, CO, USA) with DMSO or 2257 (30 nM for MDA-MB-436 and 50 nM for MDA-MB-231). Chamber slides were transferred to a humidified Chamlide stage incubator maintained at 37 °C and 5% CO_2_ on a Yokogawa spinning disk confocal microscope (Quorum Technologies, Guelph, ON, Canada) outfitted with a Hamamatsu Image EM-CCD camera. Time-lapse iDDmages at 20× magnification were collected every 4 min for 18–20 h using Volocity 6.3 software (Quorum Technologies). The time from nuclear envelope breakdown (NEBD) to anaphase, judged by the morphology of fluorescently stained DNA, was recorded for each dividing cell as previously described [14] to measure how ABCG2 perturbation affected SAC activation and its inhibition by 2257.

Multi-color Competition Assays. In vitro growth competition assays were conducted in the MDA-MB-231-Cas9+ and KPL1-Cas9+ cell lines engineered to express mCh+sgAAVS1, GFP+sgAAVS1, GFP+sgABCG2ex4^KD^, and GFP+sgABCG2ex6^KD^ to assess the relative fitness of cells with and without ABCG2 KD as previously described [17]. Briefly, each GFP+ line was mixed 1:1 with the mCh+sgAAVS1 line, and mixed cell populations were plated in 24-well plates and treated with DMSO or 50 nM 2257. The ratio of GFP+ to mCh+ positive cells was measured by flow cytometry in a fraction of cells collected at each passage. GFP+ ratios at each passage were normalized to the GFP+ ratio for each cell mixture on Day 1.

Xenograft Studies. Animal experiments were conducted in accordance with an animal use protocol approved by the University Health Network Animal Care Committee and in compliance with ARRIVE guidelines. Two million MDA-MB-231-dCas9-VPR+ cells expressing sgNTC or sgABCG2-4^OE^ were implanted into the mammary fat pad of female severe combined immunodeficiency disease (SCID) mice in a volume of 100 uL of PBS containing 50% basement membrane extract. Tumor growth was monitored regularly using caliper measurements. Once tumors reached a volume of ~200 mm^3^, sgNTC- and sgABCG2-4^OE^-injected mice were randomized into H_2_O and 2257 (6 mg/kg) treatment groups with 6–7 mice each. Mice were treated daily by oral gavage and tumor measurements were taken every 3–4 days to calculate tumor volume (L × W^2^ × 0.52) as the experimental outcome measure. All animals were humanely sacrificed once the average tumor volume of H_2_O-treated control mice reached ~1500 mm^3^ (Day 30). Statistical analyses to compare tumor volumes were conducted at this endpoint only. Tumor tissues were resected and processed to harvest protein lysates for assessing ABCG2 expression using Western blots. ABCG2 levels were quantified using ImageStudio software v5.5 and normalized to GAPDH expression.

Statistical Analyses. All statistical tests and the number of experimental replicates conducted are specified in the figure legends. Statistical analyses were performed using GraphPad Prism software v10. Two-tailed, unpaired *t*-tests were done on continuous data from multiple independent experiments assessing differences in clonogenic survival, apoptosis, Hoechst uptake (GMFI), and mitotic duration between ABCG2-perturbed and control cells. Two-tailed, unpaired *t*-tests were also used to compare GFP+ ratios between cell mixtures in individual multicolor competition assays at the final passage and to compare tumor volumes across treatment groups in xenograft experiments at endpoint only (Day 30). Error bars for all in vitro experiments present the mean ± standard deviation (SD) across independent experiments. Mean ± standard error of the mean (SEM) is presented for xenograft experiments. For all tests, *p* < 0.05 was considered significant with * *p* < 0.05, ** *p* < 0.01, *** *p* < 0.001, and **** *p* < 0.0001.

## 5. Conclusions

In summary, we identified ABCG2 as a putative in vitro-specific mechanism of resistance to the TTK inhibitor, CFI-402257, in preclinical models of TNBC. CFI-402257 demonstrated promising anti-cancer activity in clinical trials [43], and our in vivo experiments suggest its efficacy may not be limited by ABCG2/BCRP activity in patients, although ABCG2 remains worthy of consideration in the clinical context. Our findings emphasize the necessity of validating drug response mechanisms identified in cultured cells in animal models to assess their potential clinical implications and highlight that in vivo systems should be carefully tuned to recapitulate physiological conditions that influence drug response. Ultimately, the relevance of some putative mechanisms of drug resistance may require investigation in a clinical context to define their biological significance in patients.

## Data Availability

CRISPR screen data are available from the corresponding author upon reasonable request.

## Supplementary Materials

The following supporting information can be downloaded at https://www.mdpi.com/article/10.3390/ijms27062665/s1.
