# Supplementary material for: Genome-Wide CRISPR Screens Identify ABCG2-Mediated Drug Resistance to the Threonine Tyrosine Kinase (TTK) Inhibitor CFI-402257 in Breast Cancer
Source: Int J Mol Sci. 2026 Mar 14;27(6):2665. doi: 10.3390/ijms27062665 (PMC13026355; doi:10.3390/ijms27062665)
Supplement: Supplementary file 1 [file ijms-27-02665-s001.zip › SUPPLEMENTARY MATERIALS.pdf]

SUPPLEMENTARY MATERIALS

List of Supplementary Materials

Table S1. sgRNA and primer sequences

Figure S1. CRISPRa enrichment screens to identify genes whose GOF confers resistance to 2257.

Figure S2. CRISPR dropout screens to identify 2257 sensitization mechanisms in MDA-MB-231 cells

Figure S3. CRISPRa-mediated induction of ABCG2 in TNBC cell lines

Figure S4. The reported ABCG2 inhibitor, Febuxostat, does not inhibit ABCG2 efflux pump activity in breast cancer cells

Supplementary Table

Table S1. sgRNA and primer sequences

Description	Name	Sequence
CRISPRa sgRNA	hs.sgABCG2-1	GAAACCCGGGGCGCTGGGGA
CRISPRa sgRNA	hs.sgABCG2-2	GGGGAGACCCGGACATCCAG
CRISPRa sgRNA	hs.sgABCG2-3	GACATCCAGGGGACGAGCTC
CRISPRa sgRNA	hs.sgABCG2-4	GGTACCACCGCCCTCCCTCG
CRISPRa sgRNA	hs.sgABCG2-5	GGCCGGAGCGCCGAAGCACC
CRISPRa sgRNA control	sg.NTC-1	GCATAGACACGGAGTCACTG
CRISPRa sgRNA control	sg.NTC-2	GTAGCAGGAGAGACATTCAT
CRISPR KO sgRNA	hs.sgABCG2.ex4	GCTGCAAGGAAAGATCCAAG
CRISPR KO sgRNA	hs.sgABCG2.ex6	GTTGGAACTCAGTTTATCCG
CRISPR KO sgRNA control	hs.sgAAVS1	GGGGGCCACTAGGGACAGGAT
CRISPRa sgRNA	hs.sgASCL1	AAGAACTTGAAGCAAAGCGC
CRISPRa sgRNA	hs.sgNEUROD1	AGGGGAGCGGTTGTCGGAGG
CRISPRa sgRNA	hs.sgMIAT	GCGCCCATGAAATTTTAATG
CRISPRa sgRNA	hs.sgRHOXF2	CGCGTGCTCTCCCTCATCC
qPCR primer	hs.ABCG2.qPCR-F	GTTCTCAGCAGCTCTTCGGCTT

qPCR primer	hs.ABCG2.qPCR-R	TCCTCCAGACACACCACGGATA
qPCR primer	hs.ABCB1.qPCR-F	GCTGTCAAGGAAGCCAATGCCT
qPCR primer	hs.ABCB1.qPCR-R	TGCAATGGCGATCCTCTGCTTC
qPCR primer	hs.ASCL1.qPCR-F	CGCGGCCAACAAGAAGATG
qPCR primer	hs.ASCL1.qPCR-R	CGACGAGTAGGATGAGACCG
qPCR primer	hs.NEUROD1.qPCR-F	GGATGACGATCAAAAGCCCAA
qPCR primer	hs.NEUROD1.qPCR-R	GCGTCTTAGAATAGCAAGGCA
qPCR primer	hs.MIAT.qPCR-F	TGGCTGGGGTTTGAACCTTT
qPCR primer	hs.MIAT.qPCR-R	AGGAAGCTGTTCCAGACTGC
qPCR primer	hs.RHOXF2.qPCR-F	GGAGATTTAGGAAGTATGGGGTTAGTG
qPCR primer	hs.RHOXF2.qPCR-R	AAAACCTCCTCTCTTACTTTTCTACTTC
qPCR primer	hs.HPRT.qPCR-F	TGAGGATTTGGAAAGGGTGT
qPCR primer	hs.HPRT.qPCR-R	GAGCACACAGAGGGCTACAA

Supplementary Figure Legends

Figure S1. CRISPRa enrichment screens to identify genes whose GOF confers resistance to 2257. A,B) Confirmation of CRISPRa activity and target gene induction in clonally-derived dCas9-VPR+ TNBC cell lines. Stable dCas9-VPR+ cells were transfected with plasmids delivering sgRNA targeting 4 developmental genes (ASCL1, RHOXF2, MIAT, NEUROD1; as per Chavez et al, Nature Methods, 2015). Target gene expression was measured by qPCR. Relative expression was calculated by normalizing to expression in cells transfected with sgNTC-1 control. C,D) Growth curves for MDA-MB-231 and MDA-MB-436 cells over the course of the CRISPRa screens. Cells were counted at each passage to determine the cumulative number of cell doublings. E,F) Hits identified by DrugZ for CRISPRa screens conducted with additional doses in MDA-MB-231 and MDA-MB-436 cells. Analytical details are provided in the Figure 1 legend. G,H) Percentage of total sgRNA reads mapping to ABCG2-targeting sgRNA in each 2257-treated replicate at the end of the CRISPRa screens.

Figure S2. CRISPR dropout screens to identify 2257 sensitization mechanisms in MDA-MB-231 cells. A) Schematic indicating the design of CRISPR LOF screens to identify genes whose knockout sensitizes to 2257. B) Visualization of screen hits identified by the Drug Z algorithm in MDA-MB-231. For dropout screens, negative Z-scores indicate genes whose LOF is lethal in 2257 treated cells. C) Correlation between ABCG2 mRNA expression and 2257 sensitivity (i.e., area under the curve, AUC) in 590 cancer cell lines whose response to 2257 was characterized using the Broad's PRISM drug screening platform. The spearman correlation coefficient and associated p-value are indicated.

Figure S3. CRISPRa-mediated induction of ABCG2 in TNBC cell lines. Confirmation that expression of hCRISPRa-v2 library sgRNAs targeting ABCG2 (#1-5) in MDA-MB-231 and MDA-MB-436 cells stably expressing dCas9-VPR results in ABCG2 overexpression at the mRNA (A,C) and protein (B,D) levels compared to non-targeting control sgRNAs (NTC-1, NTC-2). E,F) Confirmation that sgRNAs for ABCG2 overexpression specifically induce ABCG2 and not a related ABC transporter, ABCB1. mRNA expression levels of ABCG2 and ABCB1 were assessed by qPCR in control (sgNTC) and ABCG2-overexpressing (sgABCG2-4^{OE}) cells.

Figure S4. The reported ABCG2 inhibitor, Febuxostat, does not inhibit ABCG2 efflux pump activity in breast cancer cells. A) Dose-dependent inhibition of ABCG2 by FTC. MDA-MB-231 cells were treated with serial dilutions of the ABCG2 inhibitor, FTC, and then stained with Hoechst 33342. The fluorescence intensity of Hoechst staining was measured on a spectrophotometer and normalized to DMSO control treated cells for each cell line derivative (sgNTC, sgABCG2-2^{OE} and sgABCG2-4^{OE}). Higher doses of FTC result in increased Hoechst fluorescence intensity, indicating reduced ABCG2 efflux pump activity in cells overexpressing ABCG2. B) Verification that sgABCG2-2^{OE} and sgABCG2-4^{OE} cells have reduced Hoechst staining in the DMSO-treatment condition of the FTC dose-response assay in A). C) Another reported ABCG2 antagonist, Febuxostat, does not increase Hoechst fluorescence intensity in MDA-MB-231 cells overexpressing ABCG2, indicating a lack of ABCG2 inhibition by this compound. D) Verification

that sgABCG2-2^{OE} and sgABCG2-4^{OE} cells have reduced Hoechst staining in the DMSO-treatment condition of the Febuxostat dose-response assay in C).
