# Supplementary figures and images for: Genome-Wide CRISPR Screens Identify ABCG2-Mediated Drug Resistance to the Threonine Tyrosine Kinase (TTK) Inhibitor CFI-402257 in Breast Cancer
Source: Int J Mol Sci. 2026 Mar 14;27(6):2665. doi: 10.3390/ijms27062665 (PMC13026355; doi:10.3390/ijms27062665)

# Supp. Figure S1

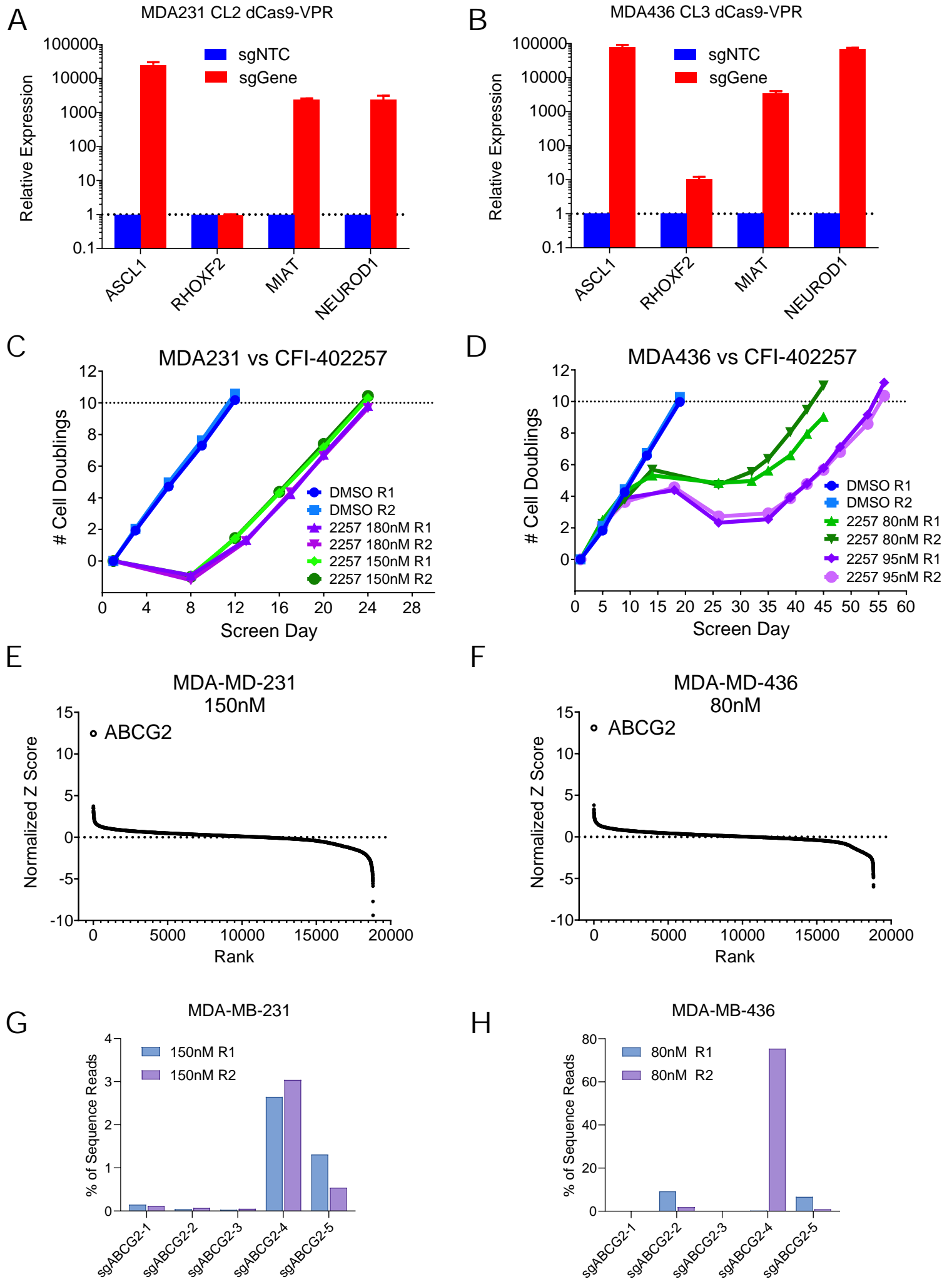

# Supp. Figure S2

A

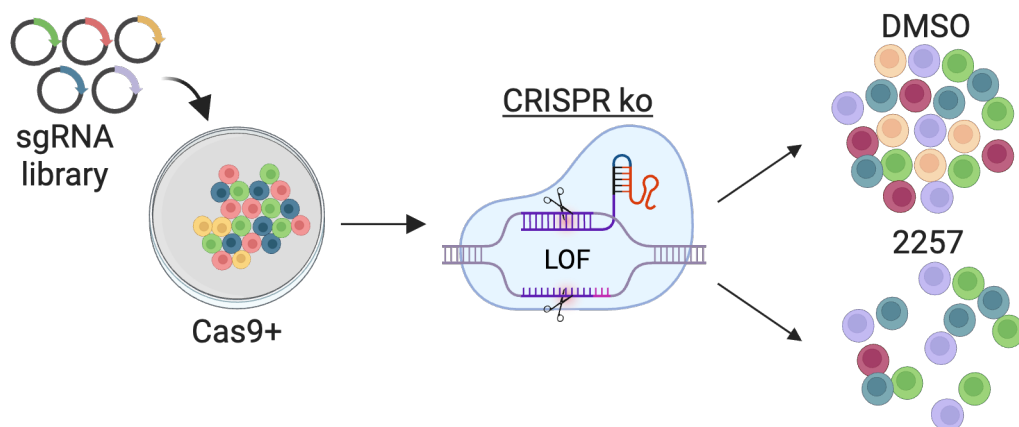

B

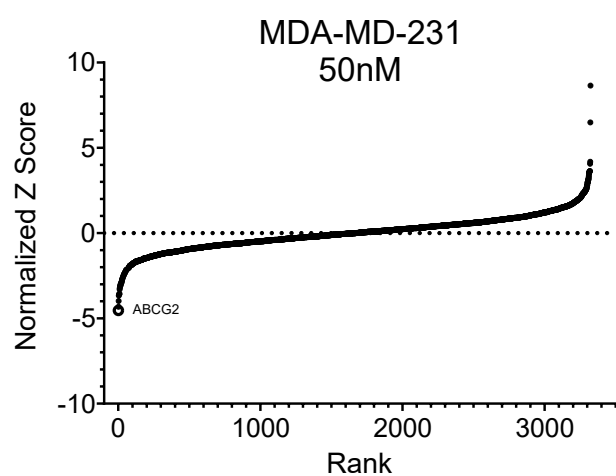

C

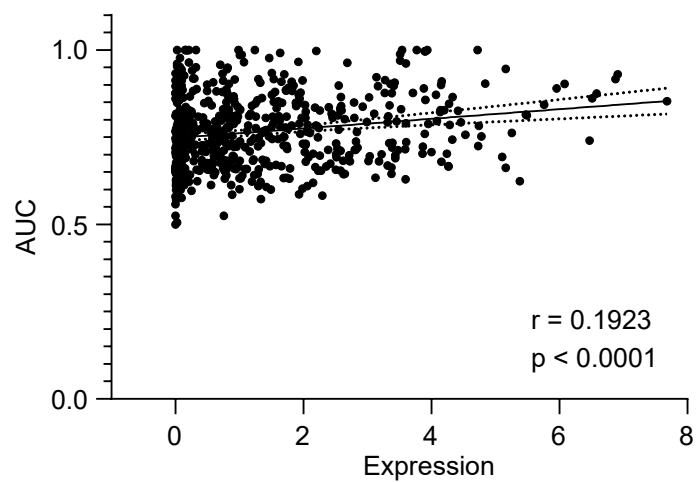

Supp. Figure S3

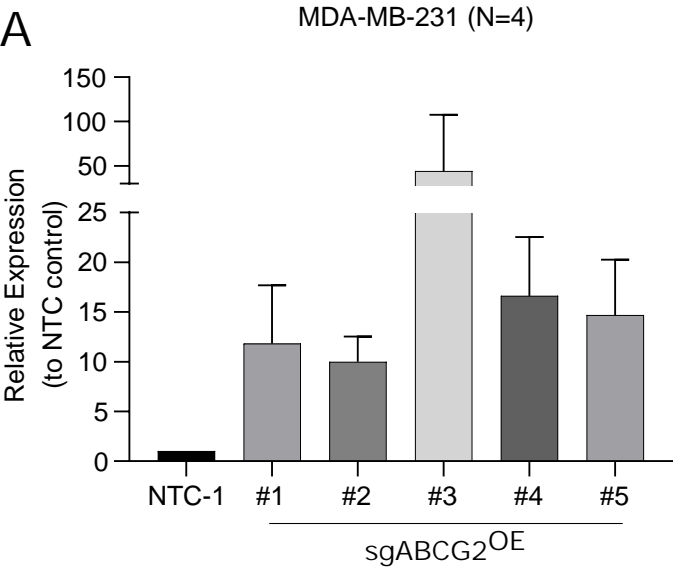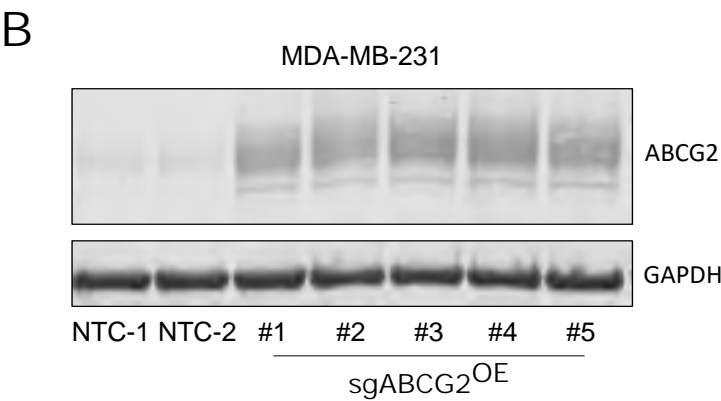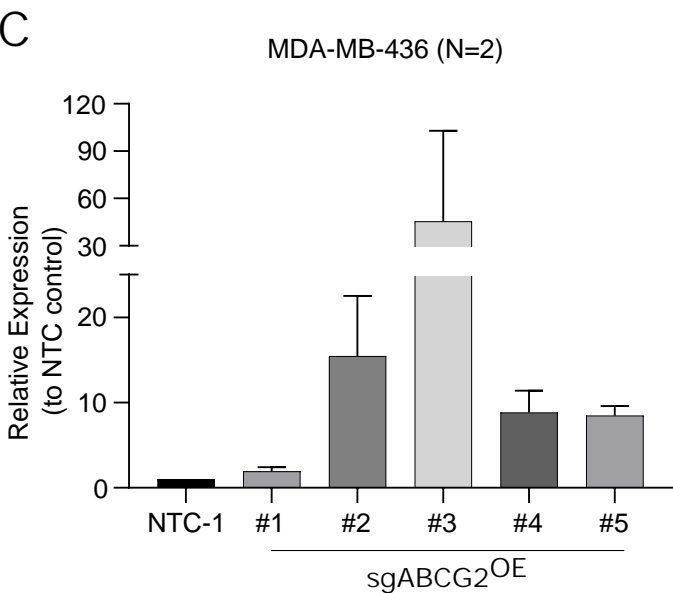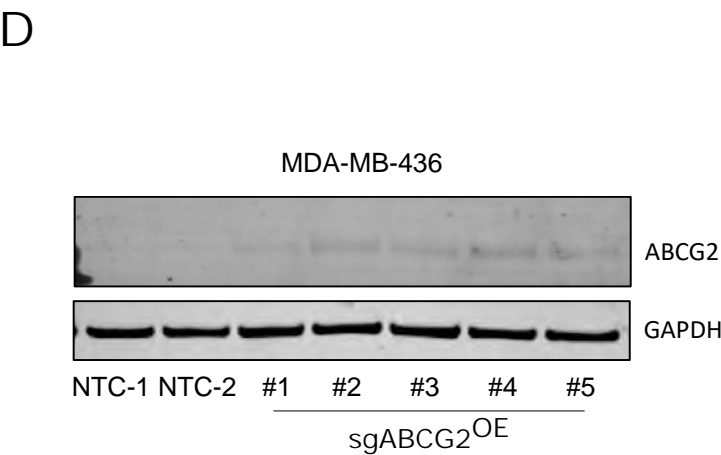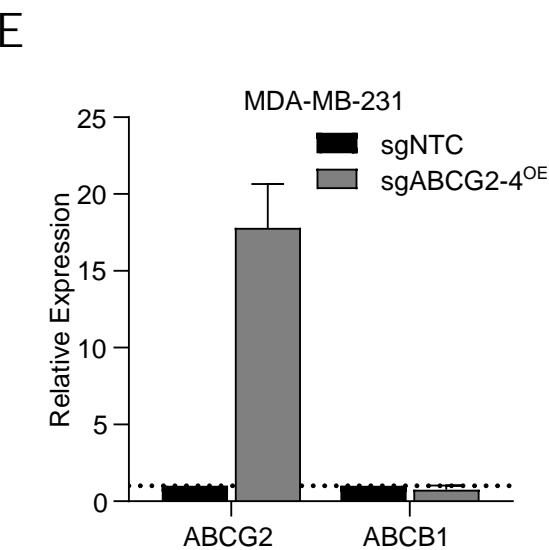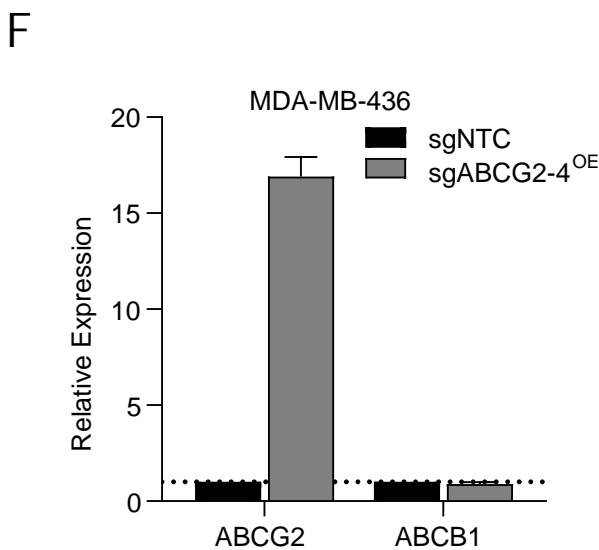

Supp. Figure S4

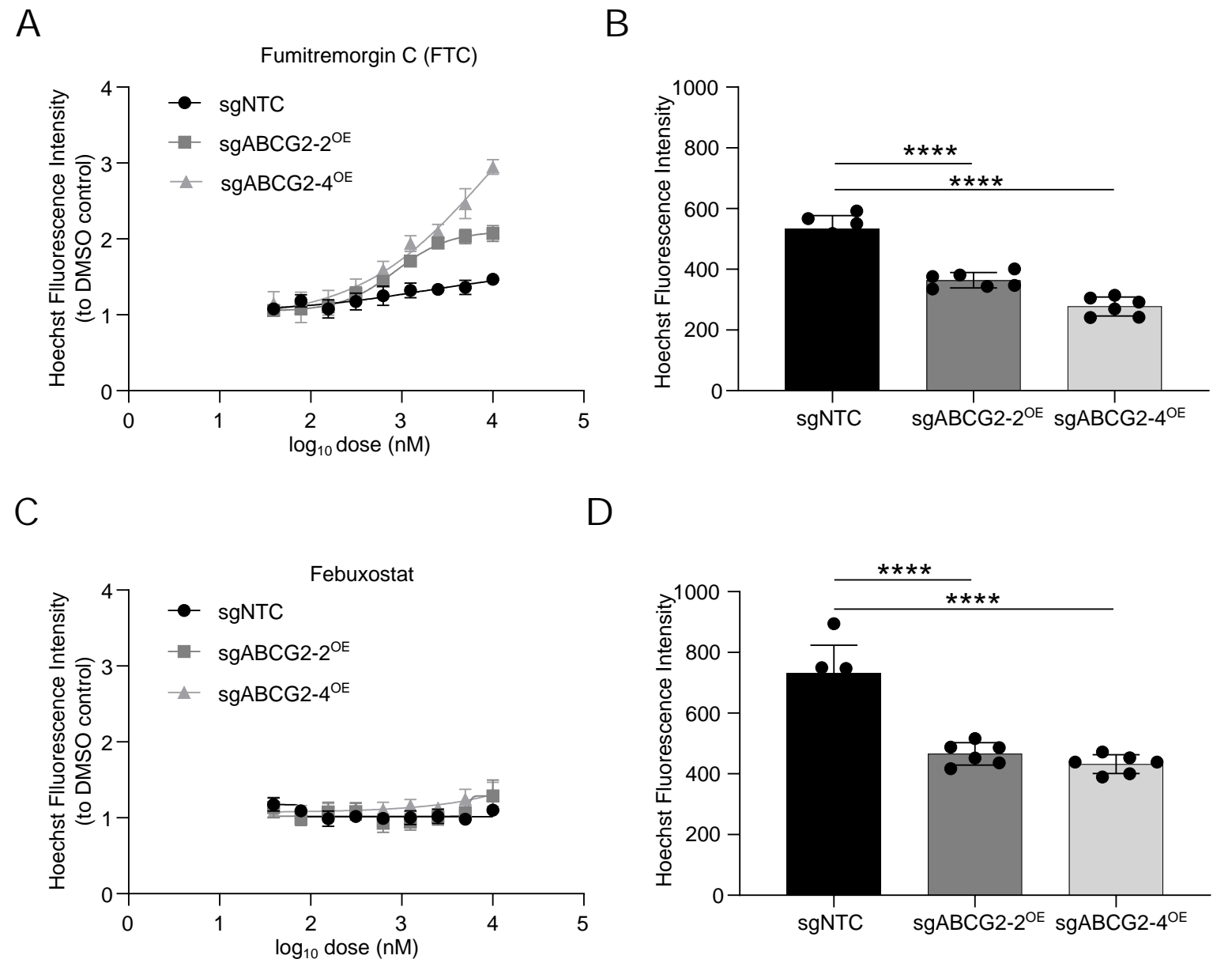

Supplement: Supplementary file 1 [file ijms-27-02665-s001.zip › 02-06-2026-SupplementaryFigures.pdf]
